# Comparative Transcriptome and Expression Profiling of Resistant and Susceptible Banana Cultivars during Infection by *Fusarium oxysporum*

**DOI:** 10.3390/ijms22063002

**Published:** 2021-03-16

**Authors:** Manoj Kaushal, George Mahuku, Rony Swennen

**Affiliations:** 1 International Institute of Tropical Agriculture (IITA), Mikocheni B, Dar es Salaam 34441, Tanzania; 2 International Institute of Tropical Agriculture (IITA), Kampala 7878, Uganda; G.Mahuku@cgiar.org; 3 International Institute of Tropical Agriculture (IITA), Arusha 447, Tanzania; R.Swennen@cgiar.org; 4 Laboratory of Tropical Crop Improvement, Division of Crop Biotechnics, KU Leuven, B-3001 Leuven, Belgium

**Keywords:** *Fusarium* wilt, banana, differentially expression gene (DEG), gene ontology (GO) annotation, Kyoto Encyclopedia of Genes and Genomes (KEGG) pathways, resistance genes

## Abstract

*Fusarium* wilt caused by *Fusarium oxysporum* f. sp. *cubense* (Foc) is one of the most destructive diseases of banana. Methods to control the disease are still inadequate. The present investigation targeted expression of defense-related genes in tissue cultured banana plantlets of *Fusarium* resistant and susceptible cultivars after infection with biological control agents (BCAs) and *Fusarium* (Foc race 1). In total 3034 differentially expressed genes were identified which annotated to 58 transcriptional families (TF). TF families such as MYB, bHLH and NAC TFs were mostly up-regulated in response to pathogen stress, whereas AP2/EREBP were mostly down-regulated. Most genes were associated with plant–pathogen response, plant hormone signal transduction, starch and sucrose metabolism, cysteine and methionine metabolism, flavonoid biosynthesis, selenocompound metabolism, phenylpropanoid biosynthesis, mRNA surveillance pathway, mannose type O-glycan biosynthesis, amino acid and nucleotide sugar metabolism, cyanoamino acid metabolism, and hormone signal transduction. Our results showed that the defense mechanisms of resistant and susceptible banana cultivars treated with BCAs, were regulated by differentially expressed genes in various categories of defense pathways. Furthermore, the association with different resistant levels might serve as a strong foundation for the control of *Fusarium* wilt of banana.

## 1. Introduction

Banana is among one of the most economically important crops in the world. Besides being a staple food crop, banana is also of critical importance for food security and income generation for small holder farmers in sub-Saharan Africa (SSA). Banana production has been negatively impacted by *Fusarium* wilt, a disease caused by the soil-borne fungus *Fusarium oxysporum* f. sp. *cubense* (Foc) [1,2]. Substantial losses in banana production have been observed around the globe [3]. The fungus persists in soil for long periods as chlamydospores even in the absence of its host. Once bananas have been infected, the fungus can easily spread through infected planting material, soil run-off and farm implements. The fungus penetrates through small openings or wounds in roots, clogs the xylem vessels and interferes with nutrient and water translocation, leading to wilting [4]. Typical wilt symptoms include yellowing of leaves, longitudinal splitting of pseudostem and necrosis [5]. Till to date no effective control exists for this disease in affected banana farms [6]. As the roots are the main organs responsible for plant growth through the assimilation of water and nutrients, biological control through root priming needs to be studied as a potential disease management strategy. Plants have inherent defense systems and banana itself also has many strategies to overcome Foc infection [7]. During Foc attack, root cells restructure and inhibit spore germination [8] significantly impacting host resistance against the phytopathogen [4]. Thus, priming of host plants with effective biocontrol agents well in advance of Foc infection could enhance plant resistance. *Bacillus* and *Trichoderma* are the two endophytes that have been successfully utilized as biological control agents (BCAs) against *F. oxysporum* in many host plants [9,10]. In addition, many defense genes associated with hormonal signaling are responsible for Foc resistance in banana [11,12,13]. Hormone signaling molecules which regulate the host immune system include abscisic acid (ABA), auxins, cytokinins, ethylene (ET), gibberellins, jasmonic acid (JA), and salicylic acid (SA) [14]. Some of these molecules such as SA, JA, and ET are widely considered to be important for plant growth [15] and in defense against biotic and abiotic stress [16]. These signal molecules induce pathogenesis-related (PR) gene expression during host–pathogen interactions [17,18]. Several defense related genes have been identified in banana roots [19], but defense gene expression of banana in response to Foc infection is inadequate. Gene expression in banana in response to Foc infection can provide important information on plant genes associated with defense to Foc. Several proteomic studies have analyzed osmotic and cold stress of banana growth and development [20,21,22]. In addition, general response of Grand Naine cultivar to Foc was also studied earlier [23]. However, examining gene expression profiles of Foc on the resistant and susceptible banana cultivars would provide more information on resistance mechanisms that could be exploited in managing *Fusarium* wilt.

The present study examined the mechanisms of banana resistance to *F. oxysporum* (Foc race 1) infection with and without biocontrol agents (*Bacillus subtilis* and *Trichoderma asperellum*). We studied the gene expression in the genetically related Foc susceptible (Mchare) and Foc resistant (Grand Naine) since Mchare (Mlali), one of the putative parents of Grand Naine. Comparative analysis was conducted to identify genes, gene ontology (GO) annotation and Kyoto Encyclopedia of Genes and Genomes (KEGG) pathways related to resistance and susceptibility of banana to Foc. In addition, metabolites profiles and expression of PR genes in response to pathogen infection were also examined. This is the first study to use biological control agents (BCAs) to study the molecular mechanisms of resistant and susceptible banana roots infected with Foc. Our results reveal the defense mechanisms for Foc resistant and susceptible cultivars with or without BCAs inoculation and serve as a strong foundation for the control of *Fusarium* wilt of banana.

## 2. Results

### 2.1. Foc Impact on Banana Plant

**Figure 1:**
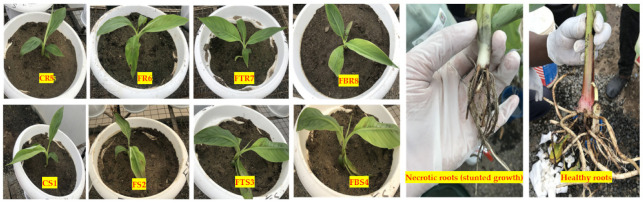
*Fusarium* symptoms in resistant and susceptible banana plantlets (field ready) after Foc infection (at four leaf stage) and biological control agents (BCAs) inoculation (after root bath, 7 and 15 days after planting). Figure also shows necrotic and stunted root growth and healthy root growth after 72 h of Foc infection in banana cultivar (Mchare). CR5 (control resistant), FR6 (*Fusarium* resistant), FTR7 (*Fusarium Trichoderma* resistant), and FBR8 (*Fusarium Bacillus* resistant) for Grand Naine and CS1 (control susceptible), FS2 (*Fusarium* susceptible), FTS3 (*Fusarium Trichoderma* susceptible), and FBS4 (*Fusarium Bacillus* susceptible) for Mchare.

We compared the symptoms on leaves as well as in roots in response to Foc inoculation in both resistant and susceptible cultivars. After 72 h of Foc inoculation, no significant symptoms were observed in leaves and roots of the resistant cultivar “Grand Naine” (CR5, FR6, FTR7, FBR8). Also, many smaller secondary and tertiary roots grew from the primary roots in all the treatments of the Foc resistant cultivar. However, the susceptible cultivar “Mchare” treated with Foc (FS2) displayed severe root necrotic symptoms and with clearly visible symptoms on the new leaves. Less necrotic symptoms were observed in the roots and leaves of the susceptible cultivar treated with BCAs (FTS3 and FBS4). Few lateral roots were observed from the main roots of the susceptible cultivar treated with BCAs (Figure 1).

### 2.2. Mapping of mRNA Sequencing Reads

We sequenced root samples from resistant and susceptible banana cultivars using BGISEQ-500 platform and generated 50.17 million reads with an average data of about 4.96 Gb per sample. Sequencing of the RNA libraries generated 49.32 to 49.71 and 49.67 to 49.74 million paired end reads for the susceptible and resistant cultivars of banana, respectively (Supplementary Table S1). Over 92% of reads for the susceptible and for the resistant cultivar were high-quality bases (quality score ≧ Q30). When mapped to the reference banana genome, on average 31.35% and 27.81% RNA-seq reads were mapped to the host banana genome of the susceptible and resistant cultivar, respectively. The uniformity of the mapping result of each sample suggests that the samples are comparable with an average unique mapping ratio of 23.21% and 20.16% for the susceptible and resistant cultivar, respectively (Supplementary Table S2). The retained high-quality pair-end reads of banana from each sample were mapped to the reference banana genome and assembled with Cuffcompare Cufflinks to reconstruct unique transcribed sequences using parameters -u, -i, -o, and -j. In total, we identified 7624 novel transcripts, 5146 of them were previously unknown splicing events for known genes, 312 of them were novel coding transcripts without any known features, and the remaining 2166 were long noncoding RNAs.

### 2.3. Detection and Annotation of DNA Polymorphisms

A total of 1,495,885 and 1,712,529 SNPs were identified in the susceptible and resistant banana cultivars, respectively. The nucleotide substitution type of SNPs also indicated higher frequency of transitions (C-T and G-A) than transversions (C-A, G-T, C-G, and T-A). Surprisingly, the ratios of transitions to transversion were equal for CS1 and CR5 (1.46), FS2 and FR6 (1.47), and FBS4 and FBR8 (1.46). The ratio of transitions to transversion for FTS3 was 1.48 and FTR7 was 1.49. It was also observed that the G-C frequency was lower than three other types of transversions (Supplementary Table S3). SNPs and INDELs of susceptible and resistant banana samples showed an identical distribution in separate genomic regions. Approximately 23% SNPs and 32% INDELs were distributed in the Up2k (upstream 2000 bp area of a gene), intergenic regions and Down2k (downstream 2000 bp area of a gene). About 77% SNPs and 68% INDELs were observed in the genic regions of the susceptible and resistant banana cultivars (Supplementary Figure S1). The most frequent alternative splicing (AS) event reported for both susceptible and resistant cultivar is SE, followed by RI and A3SS. The least frequent AS event reported for both susceptible and resistant cultivar is MXE (Supplementary Figure S2).

### 2.4. Differentially Expressed Genes Regulate Plant Responses to Foc Stress

**Figure 2:**
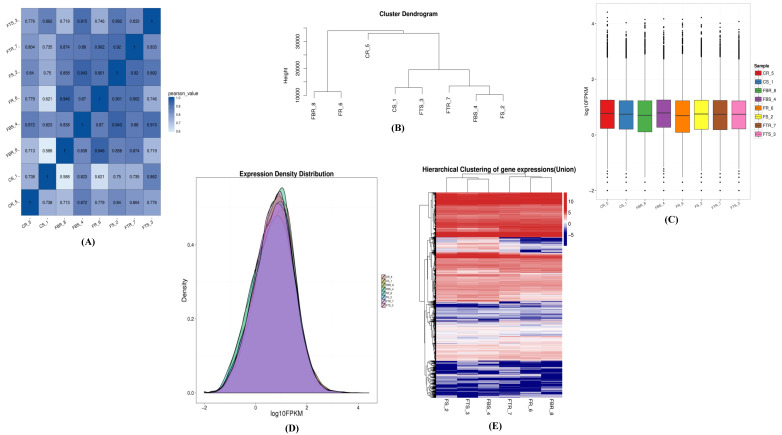
Gene expression analysis after 72 h infection of resistant (Grand Naine) and susceptible cultivars (Mchare) of banana roots. (**A**) Correlation analysis between samples. Where, the X and Y axis represent each sample. The color represents the correlation coefficient (the darker the color, the higher the correlation). (**B**) Hierarchical clustering between samples. The closer the samples, the more similar the expression level. (**C**) Gene expression Boxplot. X axis represents the sample name. Y axis represents the log10FPKM value. (**D**) Gene expression density map. X axis represents the log10FPKM value. Y axis represents the gene density. (**E**) Cluster diagram of gene expression. The gradient legend at the top right of the graph represents the FPKM value that has been logarithmically converted. Each column represents a sample, each row represents a gene, different colors represents different expression levels, red for high expression, and blue for low expression. CR5 (control resistant), FR6 (*Fusarium* resistant), FTR7 (*Fusarium Trichoderma* resistant) and FBR8 (*Fusarium Bacillus* resistant) for Grand Naine and CS1 (control susceptible), FS2 (*Fusarium* susceptible), FTS3 (*Fusarium Trichoderma* susceptible) and FBS4 (*Fusarium Bacillus* susceptible) for Mchare.

To investigate the gene expression response against Foc infection, we merged novel coding transcripts with reference transcripts to get a complete reference of both resistant and susceptible banana cultivars. Sequence profiling produced more than 396 million clean reads from all the root samples corresponding to approx. 49.5 million reads per sample (Supplementary Table S4). About 31.67%–37.47% and 38.45%–43.20% of the raw reads of the resistant and susceptible cultivars mapped to the banana reference genome (Supplementary Table S5). The number of genes that correspond to resistant and susceptible cultivars were 35,966–37,710 and 37,352–38,055, respectively (Supplementary Table S6). Sequencing data saturation analysis was used to measure whether the depth of sequencing data was sufficient for bioinformatic analysis. Increasing the number of sequenced reads increased the number of identified genes. However, when the number of sequenced reads reached a certain amount, the growth curve of identified genes flattened, indicating that the number of identified genes tends to reach a saturation (Supplementary Figure S3). In order to reflect the consistency, we calculated the Pearson correlation coefficients for all gene expression levels between the susceptible and resistant cultivars. Pearson R2 varied from 0.58 to 1.0 (Figure 2a). All the samples were hierarchically clustered by the expression level of all genes to directly reflect the relationship between compared samples for a same treatment (Figure 2b). Results revealed a more similar level of gene expression among CS1-FTS3, FS2-FBS4, and FR6-FBR8. Based on the expression information, we performed box plot to show the distribution and dispersion of the gene expression level of each sample. The box plots of the relative log10 FPKM values for each RNA-seq library displayed few distributional differences among the libraries (Figure 2c) suggesting similar transcription profiles. We further reflected the gene abundance change and the concentration of gene expression in the sample interval with a density map (Figure 2d). Genes with similar expression patterns usually have the same functional correlation. To identify genes with similar expression patterns, we clustered the 9033 genes identified by cluster software and Euclidean distance matrix for the hierarchical clustering analysis of the expressed gene and sample program at the same time (Figure 2e).

**Figure 3:**
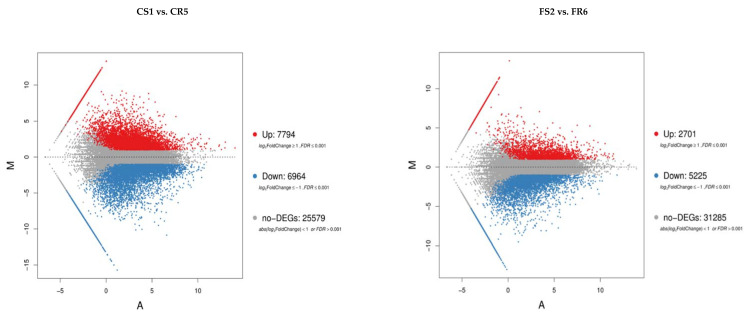

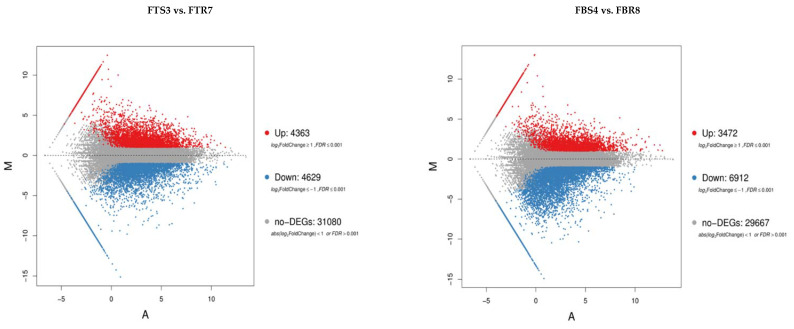
MA plot of DEGs of resistant (Grand Naine) and susceptible cultivars (Mchare) of banana roots. X axis represents value A (log_2_ transformed mean expression level). Y axis represents value M (log_2_ transformed fold change). Red dots represent up-regulated DEGs. Blue dots represent down-regulated DEGs. Gray points represent non-DEGs.

To study the gene expression of banana roots after infection with Foc, a pairwise comparison was performed between libraries to determine differentially expression genes (DEGs). On the criterion of *p* < 0.05, we analyzed and compared the same treatments of resistant and susceptible banana cultivars. Genes with FDR ≤0.05 and fold-change ≥1 were considered as differentially expressed compared with the control. We identified 14,488 (7794 upregulated and 6964 downregulated) and 7926 (2701 upregulated and 5225 downregulated) banana DEGs in control and *Fusarium* inoculated plants, respectively of the susceptible cultivar compared to the resistant one. Similarly, we detected 8992 (4363 upregulated and 4629 downregulated) and 10,384 (3472 upregulated and 6912 downregulated) banana DEGs in *Fusarium-Trichoderma* and *Fusarium-Bacillus* inoculated plants, respectively of the susceptible cultivar compared to the resistant one (Figure 3). Again, the infection responsive DEGs in the susceptible cultivar was delineated by cluster analysis.

### 2.5. GO and KEGG Function Annotation of DEGs

**Figure 4:**
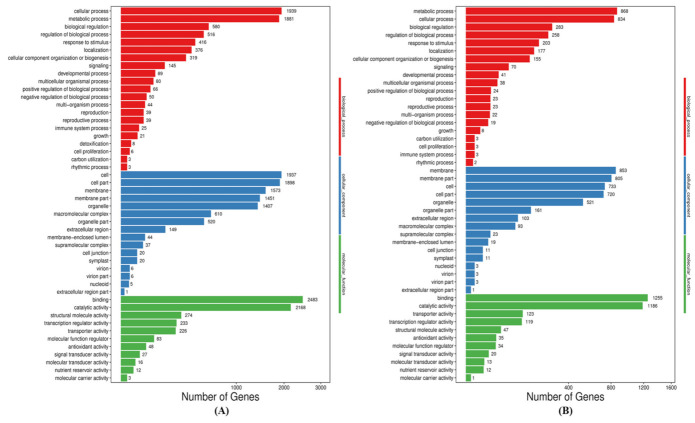

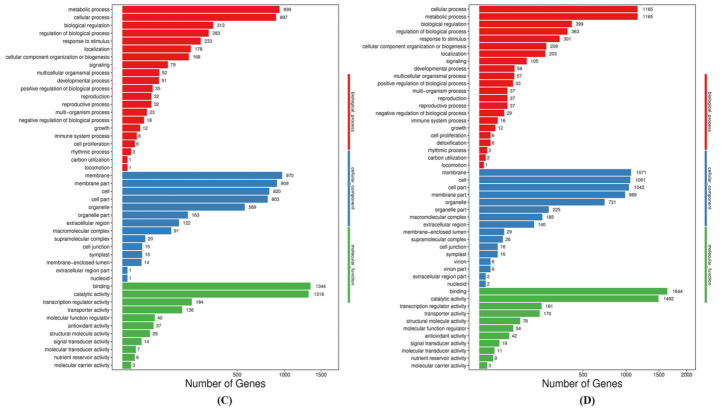
GO classification of all DEGs of resistant (Grand Naine) and susceptible cultivars (Mchare) of banana roots. X axis represents number of DEG. Y axis represents GO term. (**A**) CS1 vs. CR5, (**B**) FS2 vs. FR6, (**C**) FTS3 vs. FTR7 and (**D**) FBS4 vs. FBR8. Red: Biological process, Blue: Cellular components, Green: Molecular functions.

Gene ontology (GO) functional analysis showed that DEGs in the CS1 vs. CR5 group were enriched into 335 GO terms, of which six were significantly enriched; DEGs in the FS2 vs. FR6 group were enriched into 1005 GO terms, of which three were significantly enriched; DEGs in the FTS3 vs. FTR7 group were enriched into 1124 GO terms, of which four were significantly enriched, and DEGs in the FBS4 vs. FBR8group were enriched into 1261 GO terms, of which seven were significantly enriched (Table 1). GO analyses of all these DEGs were categorized into three categories viz. biological processes, cellular component, and molecular function (Figure 4).

**Figure 5:**
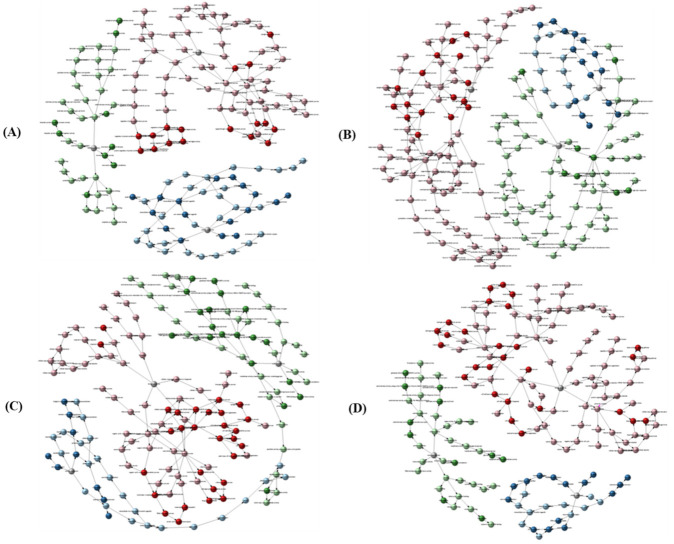
GOTerm relationship network of resistant (Grand Naine) and susceptible cultivars (Mchare) of banana roots. X axis represents number of DEG. Y axis represents GO term. Each node represents a GOterm, the different colors represent the different functional classes to which it belongs. Red: Biological process, Blue: Cellular components, Green: Molecular functions. Darkness indicates a significant enrichment (qvalue < 0.01) of GOterm, lightness indicates no significant enrichment of GOterm, gray indicates no enrichment of GOterm. (**A**) CS1 vs. CR5, (**B**) FS2 vs. FR6, (**C**) FTS3 vs. FTR7 and (**D**) FBS4 vs. FBR8.

**Figure 6:**
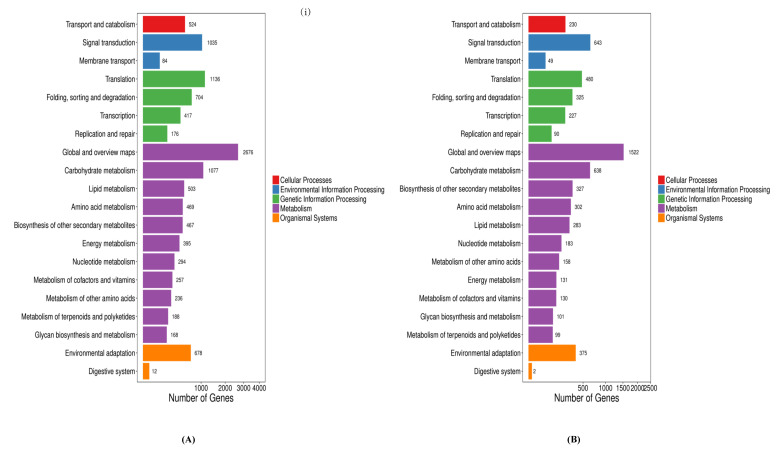

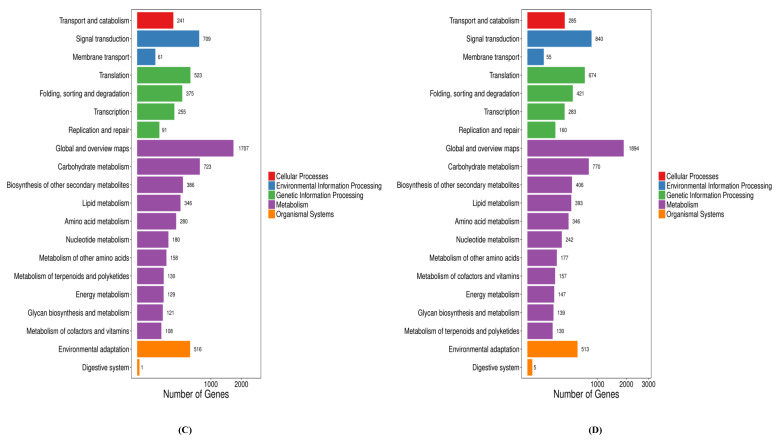

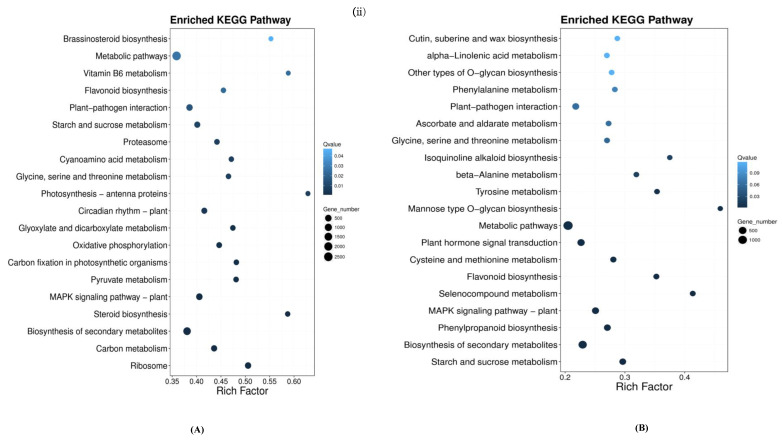

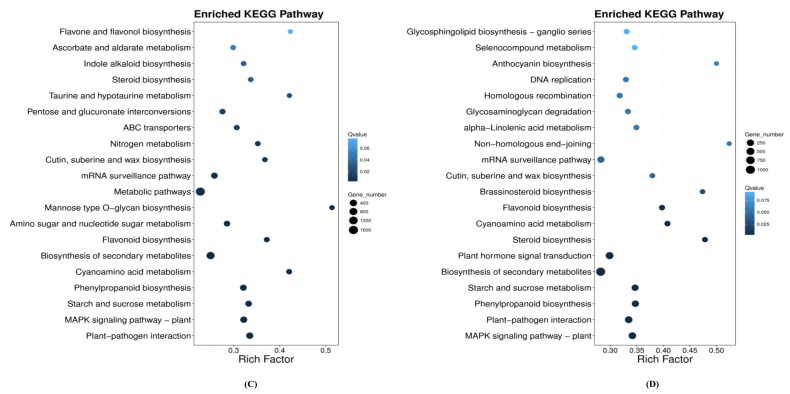
**(i) Up-** Pathway classification of DEGs of resistant (Grand Naine) and susceptible cultivars (Mchare) of banana roots. X axis represents number of DEG. Y axis represents functional classification of KEGG. **(ii) Below-** Pathway functional enrichment of DEGs. X axis represents enrichment factor. Y axis represents pathway name. The color indicates the q-value (high: White, low: Blue), the lower q-value indicates the more significant enrichment. Point size indicates DEG number (bigger dots refer to larger amounts). Rich Factor refers to the value of enrichment factor, which is the quotient of foreground value (the number of DEGs) and background value (total Gene amount). The larger the value, the more significant enrichment. (**A**) CS1-VS-CR5, (**B**) FS2-VS-FR6, (**C**) FTS3-VS-FTR7, and (**D**) FBS4-VS-FBR8.

Among the biological processes, cellular and metabolic processes were most prominent. In the cellular components, cell, cell part, membrane, membrane part, and organelle were prominently represented. Among the molecular functions, binding and catalytic activity were dominant among the GO terms in the resistant and susceptible cultivar. GO term relationship networks analysis was performed to demonstrate and confirm the roles of BCAs in the resistant and susceptible cultivar (Figure 5). We further analyzed pathway enrichment to get more insights into classification and functional enhancement in resistant and susceptible banana cultivars. The DEGs between all treated and control groups of the resistant and susceptible cultivars were enriched to 20 subclasses of 5 broad pathways (cellular processes, environmental information processing, genetic information processing, metabolism and organismal systems) analyzed utilizing the KEGG database (Figure 6 i).

KEGG enrichment analysis revealed that the DEGs in the four comparison groups were enriched in 133 (CS1 vs. CR5), and 131 (FS2 vs. FR6, FTS3 vs. FTR7 and FBS4 vs. FBR8) pathways, respectively, of which 4 (CS1 vs. CR5), 7 (FS2 vs. FR6), 5 (FTS3 vs. FTR7) and 4 (FBS4 vs. FBR8) pathways were significantly enriched. A total of 21 KEGG pathways in the four comparison groups showed significant enrichment, of which two pathways were common and significantly enriched in all the treated and control groups of the resistant and susceptible cultivar as follows: Biosynthesis of secondary metabolites and MAPK signaling pathway (Table 2). The pathways with the greatest enrichment between control plants of the resistant and susceptible cultivar were glyoxylate and decarboxylate metabolism, oxidative phosphorylation, steroid biosynthesis and carbon metabolism. The resistant and susceptible cultivar inoculated with *Fusarium* demonstrated the greatest enrichment of metabolic pathways, plant hormone signal transduction, starch and sucrose metabolism, cysteine and methionine metabolism, flavonoid biosynthesis, selenocompound metabolism, and phenylpropanoid biosynthesis. The resistant and susceptible cultivar with *Fusarium-Trichoderma* inoculation were enriched with mRNA surveillance pathway, mannose type O-glycan biosynthesis, amino acid and nucleotide sugar metabolism, cyanoamino acid metabolism and plant–pathogen interaction. Enrichment of plant hormone signal transduction, phenylpropanoid biosynthesis, starch and sucrose metabolism and plant–pathogen interaction were found in the *Fusarium-Bacillus* treated resistant and susceptible cultivar (Figure 6-ii). The differences in degree of enrichment and pathway specific enrichment suggested that responsive variability existed between the variously treated plants with BCAs and *Fusarium*. These results also suggest that these pathways and processes are involved in the *Fusarium* stress response among the different banana cultivars and treatments.

### 2.6. Transcription Factor-Encoding Genes and Co-Expression Analysis of DEGs

**Figure 7:**
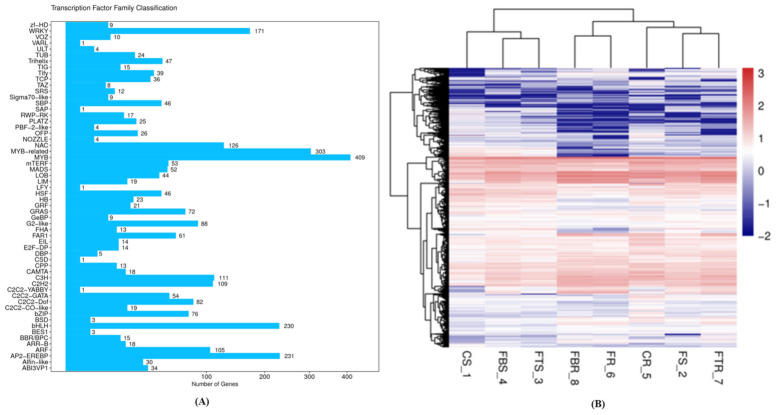
(**A**) DEGs classification on transcriptional families (TF) family of resistant (Grand Naine) and susceptible cultivars (Mchare) of banana roots. (**B**) Expression heatmap of TF coding DEGs of resistant and susceptible cultivars of banana samples.

**Figure 8:**
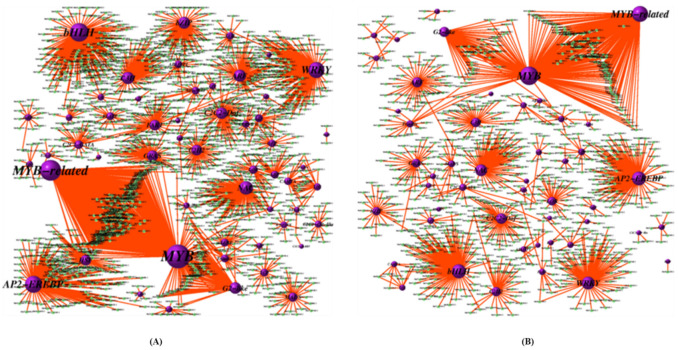

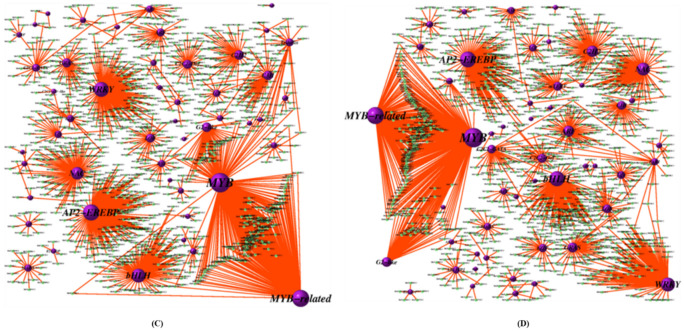
TF-DEG (differentially expressed genes) network of resistant (Grand Naine) and susceptible cultivars (Mchare)of banana roots. The red and green dots represent the up-regulated and down-regulated DEGs, respectively. Purple ball represents transcription factor, the greater the node the more DEGs the transcription factor regulate. (**A**) CS1 vs. CR5, (**B**) FS2 vs. FR6, (**C**) FTS3 vs. FTR7, and (**D**) FBS4 vs. FBR8.

To regulate gene expression, transcription factors (TF) recognize DNA in a sequence-specific manner. A total of 3034 genes were identified as DEGs and annotated in 58 different TF families for resistant and susceptible cultivars (Figure 7). In the BCA inoculated resistant and susceptible cultivars, the top largest differentially expressed TF families that were involved in response to the pathogen challenge include MYB (402 members) followed by MYB-related (303 members), AP2-EREBP (231 members), bHLH (230 members), WRKY (171 members), and NAC (126 members) (Figure 7). The expression levels of transcription factors coding DEGs in each sample of the resistant and susceptible cultivars were clustered. TF families such as MYB, bHLH, and NAC TFs were mostly up-regulated in response to pathogen stress, whereas AP2/EREBP were mostly down-regulated. Co-expression analysis were conducted using the normalized gene expression values of the common DEGs from the resistant and susceptible cultivar and Pearson correlation coefficient (r) was calculated. We used similar clusters to construct the connecting networks pairs with similarity score above 10. The TFs belonging to MYB, MYB-related, AP2-EREBP, bHLH, WRKY, and NAC TF families had the maximum number of significant edges. With other genes, many of these edges were positive that displayed conserved downregulation (Figure 8). In our network analysis TFs-MYB displayed a role in secondary metabolism and plant defense response. bHLH has been found to be crucial for plants against disease stress. This predicted interaction network of TFs in resistant and susceptible cultivars with different proteins could be useful to elaborate the DEGs role in studying the resistance mechanism in the banana host against *Fusarium*.

**Figure 9:**
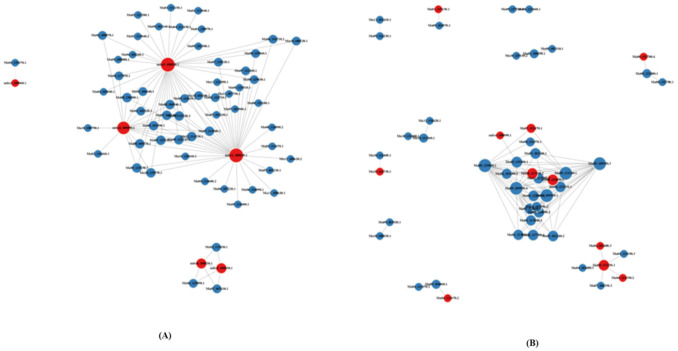

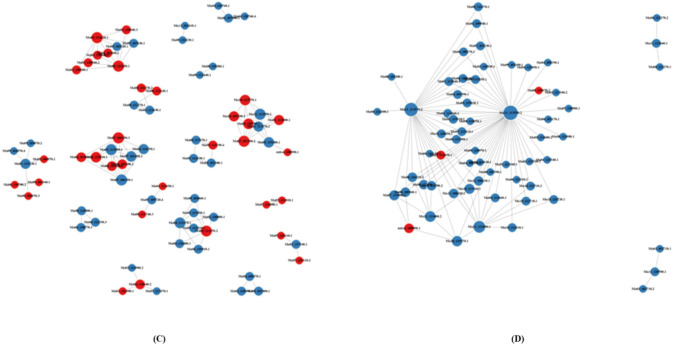
Protein–protein interaction network of resistant (Grand Naine) and susceptible cultivars (Mchare) of banana roots. The red dots refer to up-regulated genes, while the blue dots refer to down-regulated genes. The size of the circle indicates the number of interactions. (**A**) CS1 vs. CR5, (**B**) FS2 vs. FR6, (**C**) FTS3 vs. FTR7, and (**D**) FBS4 vs. FBR8.

Protein–protein interaction (PPI) network revealed a better understanding of biological processes and interconnections (Figure 9). PPI approach was used to analyze and compare the defense pathways against *Fusarium* of resistant and susceptible cultivar with and without inoculation of BCAs. We constructed PPI network of resistant and susceptible cultivars considering node degrees and found that a significant (58 and 6 (control), 40 and 11 (*Fusarium*), 41 and 33 (*Fusarium-Trichoderma*), 63 and 3 (*Fusarium-Bacillus*)) number of genes were down-regulated and up-regulated, respectively. Most of the genes in *Fusarium-Trichoderma* showed a significant up-regulation compared to other treatments. PPI network protein significantly enriched in mRNA surveillance, MAPK signaling pathway, plant–pathogen interaction and signal transduction as displayed by KEGG pathways. The results revealed the importance of splicing activity for triggering defense gene against *Fusarium* infection in banana.

### 2.7. Validation of the Selected Genes for Different Treatment Groups

The transcript levels of the top six largest differentially expressed genes (MYB, MYB-related, AP2-EREBP, bHLH, WRKY, and NAC) were validated by RT-qPCR. The expression levels of all these genes were affected by the *Fusarium* infection as compared to the susceptible and resistant control plants. The expression level of MYB gene was upregulated about 3.5 times upon BCAs inoculation. Identical results were obtained for bHLH, where however the transcript level upregulated 2.5-fold when compared to susceptible and resistant control plants. On the other hand, upregulation in the expression profile of other genes were found with BCAs treated plants but transcript level was only increased 1.5-fold when compared control.

## 3. Discussion

Unlike other crops, the defense mechanism of banana in response to pathogen infection at the molecular level is not well understood. In the present study, we used the BGISEQ-500 platform, a high throughput DNA sequencing approach for performing transcriptome sequencing to investigate changes in banana. The library was constructed from root samples of Grand Naine and Mchare cultivars that are resistant and susceptible, respectively to Foc race 1. More than 90% of the sequences from the resistant and susceptible cultivar were of high-quality with a score ≥Q30. The average mapping ratio to the reference genome was 29.58% and 37.64%, respectively. On average 29.58% reads were mapped, and the uniformity of the mapping result of each sample suggest that the samples were comparable. Functional annotation analysis revealed that 42,726 genes were expressed in all the root samples and 312 of them were novel genes.

Further, to screen the genes responsible for *Fusarium* resistance and better understand the mechanism underlying the resistance of banana to Foc race 1, we carried out a DEG analysis. The analysis has a higher precision as compared to microarrays and was used to distinguish gene expression between the resistant and susceptible cultivar giving a quantitative measure of transcript abundance [24]. The resistant cultivar contained a novel set of genes associated with numerous functions to reduce pathogen colonization. The total number of DEGs in the *Fusarium* resistant “Grand Naine” was far less than that in *Fusarium* susceptible “Mchare” (Figure 3). This might be due to the higher susceptibility of micropropagated young banana plantlets [25,26].

### 3.1. Transcription Factors

Transcription factors (TFs) regulate gene expression with regard to plant defense against various phytopathogens, thus are crucial in plant development. In the present study, most of the TF families showed heterogenous expression profiles with complex regulatory behavior during disease stress. 58 TF families were identified, among which MYB, MYB-related, AP2-EREBP, bHLH, WRKY, and NAC were the most abundant and involved in various stress responses [27] in all compared groups. Among these TF types, the most abundant were NAC with 126 members (126), and MYB with 409 (Figure 7A). Upregulated TFs, including MYB, bHLH and NAC were found in the Foc resistant cultivar “Grand Naine”. SpMYB TF expression was also significantly induced in *Arabidopsis* challenged with *Fusarium oxysporum* [28]. WRKY22, WRKY33, and DREB TFs showed a different expression pattern between *Fusarium* resistant and susceptible banana cultivars, and were differentially expressed in the CS1 vs. CS5, FS2 vs. FR6, FTS3 vs. FTR7 and FBS4 vs. FBR8 group. The expression levels of WRKY22, WRKY33, and DREB were two-fold higher in the resistant cultivar “Gand Naine” than in “Mchare” under untreated conditions. Bai et al. [26] also suggested that three WRKY TFs showed different expression patterns in Cavendish banana cultivars after Foc infection.

A large family of plant-specific TFs encoded by MYB gene family plays a crucial role in stress regulation followed by overall plant development. Isolated from *Zea mays*, the first plant MYB gene, C1, was involved in anthocyanin biosynthesis [29]. In comparison, our study found a total of 409 (MYB) and 303 (MYB-related) proteins in banana roots. Specifically, MYB and 303 MYB-related, were involved in plant–pathogen pathways, and differentially expressed in the CS1 vs. CS5, FS2 vs. FR6, FTS3 vs. FTR7, and FBS4 vs. FBR8 group. These are involved in many functions including secondary metabolism, gene regulation and response to stress (Figure 7 and Figure 8). MYB and MYB-related proteins form transcription complexes and work along with bHLH proteins in various cellular processes including cell cycle regulation [30].

The bHLH proteins are one of the largest TF families. These proteins regulate various biological processes including wound and drought stress responses, hormone signaling and tissue development in plants [31]. Our analysis showed that 230 bHLH proteins were expressed in banana roots, and differentially expressed in the CS1 vs. CS5, FS2 vs. FR6, FTS3 vs. FTR7 and FBS4 vs. FBR8 group (Figure 7 and Figure 8). The bHLH proteins were classified into 32 subfamilies based upon evolutionary and genome analysis having the same plant subfamilies with identical functionalities [32]. These bHLH subfamilies were found to be involved in the control of biosynthesis of phenylpropanoid, flavonoid and anthocyanin [33].

The NAC domain-containing 126 protein has been reported to be involved in resistance of banana to Foc [34], and differentially expressed in the CS1 vs. CS5, FS2 vs. FR6, FTS3 vs. FTR7 and FBS4 vs. FBR8 group. Other studies also reported that it is involved in the susceptibility of plants to diseases with NAC21/22 involvement in wheat (*Triticum aestivum*) [35] and reduced bacterial wilt resistance due to the over-expression of SmNAC [36] suggesting the importance of plant NAC in plant–pathogen interaction. A recent study by Dong et al. [37] revealed that six NAC TFs were up regulated in the Foc1 vs. CK group and Foc1 vs. Foc4 group, respectively, but not differently expressed in the Foc4 vs. CK group.

C3H are the zinc finger families of proteins involved in RNA-binding activity in pre-mRNA processing, transcriptional regulation and tolerance to adverse stress conditions [38]. In total 111 root specific C3H proteins were revealed in banana roots. On the other hand, 68 and 67 C3H family genes were identified in *Arabidopsis* and rice, respectively [39]. Our study revealed that C3H proteins, are differentially expressed in the CS1 vs. CS5 and FS2 vs. FR6 group. Other zinc finger families were found in banana including WRKY and C2H2 related genes and differentially expressed in the FTS3 vs. FTR7 and FBS4 vs. FBR8 groups (Figure 7 and Figure 8).

In the present study, WRKY TFs which are well-established regulators of defense genes were expressed more and upregulated in the FTS3 vs. FTR7 and FBS4 vs. FBR8 groups (Figure 7 and Figure 8). WRKY is a crucial TF involved in both basal and induced resistance of banana to Foc. WRKY50 involvement is also well-known in the signaling pathway of salicylic acid in *Arabidopsis* [40].

All candidate genes (MYB, MYB-related, AP2-EREBP, bHLH, WRKY, and NAC) were tested using RT-qPCR assay to evaluate their influence on the gene expression in *Fusarium infected* and BCAs inoculated plants. MYB genes, the largest families in plants play a key role in development such as two displayed in our study- secondary metabolism and plant defense response. bHLH has been found to be crucial for plant growth including photomorphogenesis, light signal transduction, and in plant response to disease stress.

### 3.2. Comparative GO and KEGG Analysis

We utilized comparative transcriptome sequencing approach to identify DEGs in banana root samples under Foc stress conditions and BCAs inoculation. GO annotation and KEGG pathway analysis revealed the DEGs enrichment in multiple GO terms and pathways including plant–pathogen interaction, MAPK signaling, starch and sucrose and cyanoamino metabolism and biosynthesis of phenylpropanoid and secondary metabolites in BCAs treated plants (FTS3 vs. FTR7 and FBS4 vs. FBR8 groups). These pathways were associated with transcription, metabolism, signal transduction and defense, and transport, which are involved in the regulation of responses to pathogen attack [41]. Multiple pathways against Foc infection were found through the genes that are involved in the plant–pathogen interaction pathway [42]. Plant responses to pathogen attack also occur through MAPK signaling that mainly include expression of pathogenesis-related (PR), hypersensitive reaction (HR), and cell wall lignification which further activated the immune response. The involvement of the amino acid metabolism in plant resistance to diseases was recently reported. For instance, a strong up-regulation of asparagine synthetase was observed in infected wild-type tomato and in *Botrytis cinerea*–tomato interaction [43]. The aspartate and aspartate-derived amino acids content affected the defense responses in *Arabidopsis thaliana* due to the over-expression of cytosolic aspartate amino transferase [44]. In addition, the plant hormone signal transduction pathway was found only in cultivars treated with BCAs (FBS4 vs. FBR8 group). The role of plant hormones in response to plant defense against biotic stress has been well established [45,46]. Similarly, to other species [47], our data displayed very important roles of secondary metabolites in plant pathogen defense. Foc infected and BCAs inoculated banana cultivars were found with upregulated genes in the FTS3 vs. FTR7 and FBS4 vs. FBR8 groups. These enzymes play a major role in antifungal compound synthesis and other components of the cell wall that are involved in the phenylpropanoid pathway and other pathways. These results indicate that these pathways were related to Foc stress response in banana. Furthermore, enrichment pathways and DEGs suggested the contrasting response mechanisms against Foc stress under varied treatments. In the present study, MYB (409 genes) transcription factors were also differentially expressed in Foc inoculated plants. In *Arabidopsis*, MYB transcription factor was found to respond to stress [48]. In addition, we observed that the genes encoding MYB-related, AP2-EREBP, bHLH, WRKY, NAC, and C3H were differentially expressed in the FTS3 vs. FTR7 and FBS4 vs. FBR8 groups (Figure 7). These TF are involved in diverse biological processes and contributed to Foc stress response through modulation of plant immune responses.

### 3.3. Defense Response Genes

Production of pathogenesis-related (PR) proteins have been widely studied in plant pathogen interactions. In our study, the constitutive expression of glucanases and chitinases was significantly higher in the resistant cultivar “Grand Naine” compared with the susceptible cultivar “Mchare”. When inoculated with the Foc and respective BCAs, expression of pathogenesis related (PR)-1, glucanases and chitinases were affected in both resistant and susceptible cultivars. PR-1 was up-regulated in the resistant CR1, FR6, and FRT7 and down-regulated in the susceptible cultivars. The expression of glucanase changed dramatically over time in FS2 and FBS4. Very high expression of glucanase genes were observed in the resistant cultivar “Grand Naine” compared with those in the susceptible cultivar “Mchare” where it was observed only at the stages of Foc infection and BCAs inoculation. Upregulation of chitinase was found in resistant cultivar “Grand Naine” while those in the susceptible cultivar “Mchare” showed up-regulation only during the BCAs inoculations (FTS3 and FBS4). Higher accumulation of mRNA of PR-1, glucanases and chitinases was observed in resistant and susceptible banana cultivars after BCAs inoculation and Foc infection; suggesting their involvement in plant defense response in bananas. The findings identified some Foc stress related genes through comparative transcriptome approaches that can be harnessed for the genetic improvement of banana.

## 4. Materials and Methods

### 4.1. Plant Materials, BCAs, and Foc Inoculation

Two banana cultivars, Mchare and Grand Naine, susceptible and resistant to *F. oxysporum* (Foc race 1), respectively, were used in this study. Tissue-culture derived plantlets of the two cultivars were procured from Crop Biosciences Limited, Arusha, Tanzania. Two indigenous strains, viz. *Bacillus subtilis* and *Trichoderma asperellum* were used as endophytic BCAs. *F. oxysporum* f. sp. *cubense* (Tropical Race 1) was isolated and identified from the pseudostem of diseased plants of farmer’s field in Kilimanjaro region, Tanzania and preserved in the laboratory [45,46]. Roots of banana plantlets were dipped in a 10-mL solution of *Bacillus subtilis* (1 × 10^6^ cfu/mL) and *Trichoderma asperellum* (1 × 10^5^ cfu/mL) inoculation for 30 min before planting in pots. Control plantlets were treated with sterile distilled water. Treated plantlets were transplanted into pots (with 7 kg capacity) containing 5 kg sterile sand and soil (loamy textured) mixed in the ratio of 2:1. A week after planting, plantlets were drenched with their respective treatment solutions at @5% *v*/*v*. The treatment was repeated 15 days later. Plantlets were grown in the greenhouse at 30 ± 2 °C (day)/25 ± 2 °C (night) with ~14 h photoperiod. With some modifications, banana plantlets were inoculated with Foc according to Van et al. [19]. At the four leaf stage, roots of banana plantlets were washed with sterile distilled water and wounded by gentle crushing, and dipped in a beaker containing 200 mL of 10^6^ conidia/mL spore suspension of Foc for 30 min. Similarly, the control plants were treated with sterile distilled water. After treatment, plants were replanted in pots as described above. This sample set up was done in triplicates. Root samples were collected after 72 h of Foc or sterile distilled water treatment. The samples were marked as CS1 (control susceptible), FS2 (*Fusarium* susceptible), FTS3 (*Fusarium-Trichoderma* susceptible) and FBS4 (*Fusarium-Bacillus* susceptible) for Mchare and CR5 (control resistant), FR6 (*Fusarium* resistant), FTR7 (*Fusarium-Trichoderma* resistant) and FBR8 (*Fusarium-Bacillus* resistant) for Grand Naine. The samples were immediately kept in RNA later solution and transported to laboratory for further use.

### 4.2. RNA Purification, Sequencing, and Genome Mapping

Total RNA was extracted from banana roots using Agilent 2100 Bioanalyzer (Agilent RNA 6000 Nano Kit). The concentration, integrity and yield of total RNA were determined using Agilent’s 2100 Bioanalyzer with the Plant RNA Nano chip assay (Agilent Technologies, Santa Clara, CA, USA). Concentration (ng/µl) of RNA was measured by comparing with a standard sample. We analyzed RNA integrity using two methods: The ratio of the large (28S) to small (18S) ribosomal RNA subunits (28S/18S) and the RNA integrity number (RIN). RNA purity was determined on a NanoDropTM.

The poly-A containing mRNA molecules were purified using poly-T oligo attached magnetic beads. Following purification, the mRNA was fragmented into small pieces using divalent cations under elevated temperature. The cleaved RNA fragments were copied into first strand cDNA using reverse transcriptase and random primers. This was followed by second strand cDNA synthesis using DNA Polymerase I and RNase H. These cDNA fragments have the addition of a single “A” base and subsequent ligation of the adapter. The products were then purified and enriched with PCR amplification. We quantified the PCR yield by Qubit and pooled samples together to make a single strand DNA circle (ssDNA circle), which gave the final library. DNA nanoballs (DNBs) were generated with the ssDNA circle by rolling circle replication (RCR) to enlarge the fluorescent signals at the sequencing process. The DNBs were loaded into the patterned nanoarrays and pair-end reads of 100 bp were read on the BGISEQ-500 platform. Prior to assembly of sequences into scaffolds, we filtered the low-quality reads (more than 20% of the bases qualities are lower than 10), reads with adaptors and reads with unknown bases (N bases more than 5%) to get the clean reads [49]. Then we mapped those clean reads onto the reference genome. We used HISAT (Hierarchical Indexing for Spliced Alignment of Transcripts) to do the mapping step [50].

### 4.3. Novel Transcript Prediction

We used StringTie [51] to reconstruct transcripts and used Cuffcompare Cufflinks [52] tools to compare reconstructed transcripts to reference annotation. We selected “u” (Unknown, intergenic transcript), “i” (A transfrag falling entirely within a reference intron), “o” (Generic exonic overlap with a reference transcript) and “j” (Potentially novel isoform (fragment): At least one splice junction is shared with a reference transcript class code types as novel transcripts. CPC [53] was used to predict coding potential of novel transcripts and merged coding novel transcripts with reference transcripts to get a complete reference, and base of downstream analysis. The transcriptome assembly were assessed based on this reference [54].

### 4.4. Identification, Analysis of Variations, and Splicing Gene Detection

After genome mapping, we used GATK [55] to call SNP and INDEL for each sample. The unreliable sites were filtered to get the final SNP and INDEL. For the detection of differentially splicing genes (that is differential isoform relative abundance between samples), rMATS were used [56] to calculate the inclusion isoform and skipping isoform. The statistical model of MATS calculates the *p*-value and false discovery rate (FDR) that gives the difference in the isoform ratio of a gene between two conditions. In our study, gene with FDR ≤ 0.05 was defined as a significant differentially splicing gene (DSG).

### 4.5. Gene Expression and Cluster Analysis

Clean reads were mapped to reference using Bowtie2 [57], and gene expression level was calculated using RSEM [58]. Pearson correlation were analyzed between all samples. Hierarchical clustering between all samples was accomplished using hclust, ggplot2 with functions of R used for diagrams. The clustering results were displayed with javaTreeview [59] using cluster [60,61] software to analyze the expressed genes and sample scheme using Euclidean distance matrix.

### 4.6. Annotation and Functional Prediction of DEG

We detected DEGs with PossionDis based on the Poisson distribution [62]. With the GO annotation result, we classified DEGs and performed GO functional enrichment using phyper in R. The false discovery rate (FDR) was calculated for each *p* value. Similar steps were repeated with KEGG annotation results for pathway analysis of DEG. We used getorf to find ORF of each DEG. ORF were aligned to TF domains (from PlntfDB) using hmmsearch [63].

### 4.7. Quantitative PCR Validation to Confirm Expression Levels of Genes

The selected reference genes, including the most expressed genes, were validated for for qPCR assays in different samples (CS1 vs. CS5, FS2 vs. FR6, FTS3 vs. FTR7 and FBS4 vs. FBR8) groups which controls the defense patways. The 2^−∆∆ct^ method was employed to calculate the relative expression level of the targeted genes.

### 4.8. Phylogenetic Relationship and Disease Resistant Gene Prediction

We used DIAMOND [64] to map the DEGs to the STRING [65] database to obtain the interaction between DEG-encoded proteins using homology with known proteins. The top 100 interaction networks were selected and used to draw the picture. We used DIAMOND [66] to map the DEGs to the PRGdb [67] database for the detection of plant disease resistance genes based on the query coverage and identity requirement [68].

## 5. Conclusions

This is the first study to perform transcriptome and expression profile sequencing of a *Fusarium* resistant and susceptible cultivar following inoculation with BCAs. BCAs reduced the necrotic symptoms in the roots and leaves of the susceptible cultivar (FTS3 and FBS4). Results revealed DEGs that are involved in complex defense pathways including transcription, metabolism, secondary metabolites, signal transduction and defense, and transport, in response to the pathogen. The results also showed that TF families that were involved include MYB, MYB-related, AP2-EREBP, bHLH, WRKY, and NAC suggesting that these TF play important roles in response to *Fusarium* infection of banana. The expression patterns exhibited by *Fusarium*-responsive genes in the resistant and susceptible cultivar provides useful information of the molecular basis of defense in banana. These top regulatory and signaling *Fusarium*-responsive genes will allow us to explore their function and crosstalks of complex signaling networks. Furthermore, mechanistic insights gained in banana to stress response provide important clues to explore the orthologous genes and potential candidates for the development of stress tolerant banana cultivars.

## Figures and Tables

**Table 1:** Statistics of gene ontology (GO) terms with significant enrichment of differentially expression genes (DEGs) in four compared groups.

Category	GO ID	Description	Gene Ratio	pva	padj
**CS1 vs. CR5**					
MF	GO:0005840	ribosome	255/3114	0.00	0.01
MF	GO:1990904	ribonucleoprotein complex	300/3114	0.00	0.01
BP	GO:0043228	non-membrane-bounded organelle	385/3114	0.00	0.01
BP	GO:0043232	intracellular non-membrane-bounded organelle	385/3114	0.00	0.13
CC	GO:0030529	intracellular ribonucleoprotein complex	300/3114	0.00	0.01
CC	GO:0032991	macromolecular complex	610/3114	0.00	0.01
**FS2 vs. FR6**					
MF	GO:0005975	carbohydrate metabolic process	173/1245	0.00	0.01
MF	GO:0019752	carboxylic acid metabolic process	108/1245	0.00	0.01
BP	GO:0032774	RNA biosynthetic process	175/1245	0.00	0.01
**FTS3 vs. FTR7**					
MF	GO:0005975	carbohydrate metabolic process	191/1340	0.00	0.01
MF	GO:0071554	cell wall organization or biogenesis	100/1340	0.00	0.01
BP	GO:0032774	RNA biosynthetic process	222/1340	0.00	0.01
BP	GO:0006950	response to stress	127/1340	0.00	0.01
**FBS4 vs. FBR8**					
MF	GO:0006351	transcription, DNA-templated	260/1659	0.00	0.01
MF	GO:0097659	nucleic acid-templated transcription	260/1659	0.00	0.01
BP	GO:0018130	heterocycle biosynthetic process	312/1659	0.00	0.01
BP	GO:2001141	regulation of RNA biosynthetic process	200/1659	0.00	0.01
BP	GO:0051171	regulation of nitrogen compound metabolic process	223/1659	0.00	0.01
BP	GO:0060255	regulation of macromolecule metabolic process	229/1659	0.00	0.01
BP	GO:0010468	regulation of gene expression	209/1659	0.00	0.01

Where, MF: Molecular functions; BP: Biological processes; CC: Cellular functions.

**Table 2:** Distribution of DEGs in significantly enriched KEGG pathways in four compared groups.

Pathway ID	Pathway Name	CS1 vs. CR5	FS2 vs. FR6	FTS3 vs. FTR7	FBS4 vs. FBR8
		No. of DEGs	No. of DEGs	No. of DEGs	No. of DEGs
ko04016	MAPK signaling pathway-plant	469	290	372	310
ko01110	Biosynthesis of secondary metabolites	1387	836	910	1023
ko00630	Glyoxylate and decarboxylate metabolism	93	/	36	/
ko00190	Oxidative phosphorylation	150	/	/	/
ko00100	Steroid biosynthesis	54	18	31	44
ko01200	Carbon metabolism	300	/	/	/
ko01100	Metabolic pathways	/	1719	/	/
ko04075	Plant hormone signal transduction	/	374	/	493
ko00500	Starch and sucrose metabolism	/	206	/	241
ko00270	Cysteine and methionine metabolism	/	87	/	/
ko00941	Flavonoid biosynthesis	/	55	/	/
ko00450	Selenocompound metabolism	/	43	23	36
ko00940	Phenylpropanoid biosynthesis	/	226	/	290
ko03015	Mrna surveillance pathway	/	/	252	/
ko00515	Mannose type O-glycan biosynthesis	18	17	19	14
ko00520	Amino acid and nucleotide sugar metabolism	/	/	155	/
ko00460	Cyanoamino acid metabolism	/	39	66	/
ko04626	Plant–pathogen interaction	/	/	395	395

## Data Availability

The data that support the findings of this study are available from the corresponding author upon reasonable request.

## Supplementary Materials

The following are available online at https://www.mdpi.com/1422-0067/22/6/3002/s1.
