# Supplementary material for: Comparative Transcriptome and Expression Profiling of Resistant and Susceptible Banana Cultivars during Infection by Fusarium oxysporum
Source: Int J Mol Sci. 2021 Mar 16;22(6):3002. doi: 10.3390/ijms22063002 (PMC7999991; doi:10.3390/ijms22063002)
Supplement: Supplementary file 1 [file ijms-22-03002-s001.pdf]

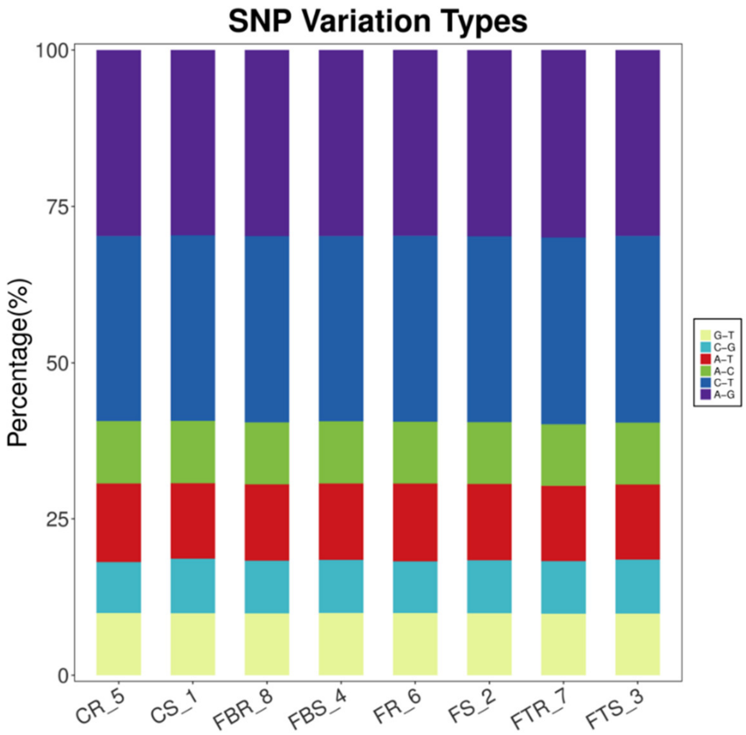

Supplementary Figure 1. SNP variant type distribution of susceptible and resistant banana cultivars. X axis represents the type of SNP. Y axis represents the number of SNP. CS1 (control susceptible), FS2 (*Fusarium* susceptible), FTS3 (*Fusarium Trichoderma* susceptible) and FBS4 (*Fusarium Bacillus* susceptible) for Mchare and CR5 (control resistant), FR6 (*Fusarium* resistant), FTR7 (*Fusarium Trichoderma* resistant) and FBR8 (*Fusarium Bacillus* resistant) for Grand Naine.

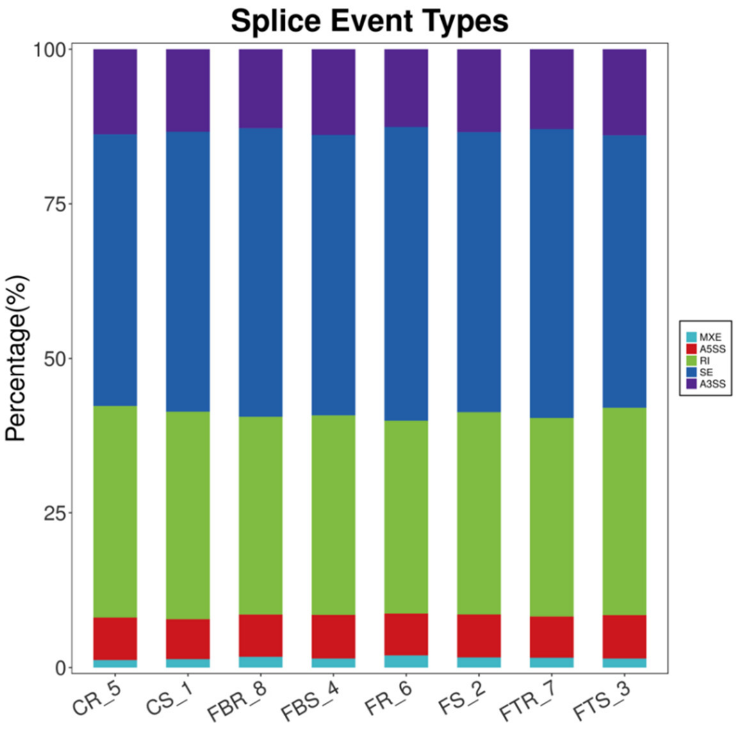

Supplementary Figure 2. Splice event types of susceptible and resistant banana cultivars. X axis means the type of splicing. Y axis means the amount. Different columns represent different splicing events. CS1 (control susceptible), FS2 (*Fusarium* susceptible), FTS3 (*Fusarium Trichoderma* susceptible) and FBS4 (*Fusarium Bacillus* susceptible) for Mchare and CR5 (control resistant), FR6 (*Fusarium* resistant), FTR7 (*Fusarium Trichoderma* resistant) and FBR8 (*Fusarium Bacillus* resistant) for Grand Naine.

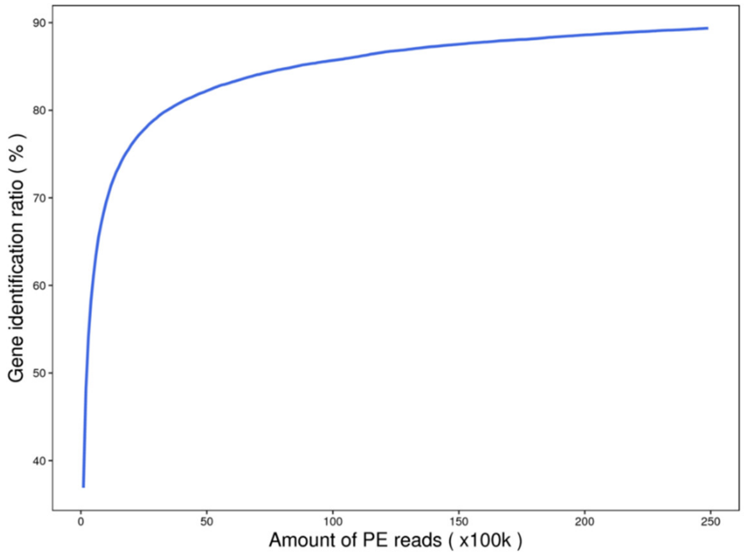

Supplementary Figure 3. Sequencing saturation of susceptible and resistant banana cultivars. The X axis represents the number of clean reads, in units of 100k; the Y axis indicates the number of detected genes, in units of %.

Supplementary Table 1. Clean reads quality metrics

Sample	Total Raw Reads (M)	Total Clean Reads (M)	Total Clean Bases (Gb)	Clean Reads Q20 (%)	Clean Reads Q30 (%)	Clean Reads Ratio (%)
CR_5	50.17	49.67	4.97	98.33	92.46	99.01
CS_1	50.17	49.57	4.96	98.36	92.78	98.81
FBR_8	50.17	49.71	4.97	98.22	92.35	99.09
FBS_4	50.17	49.32	4.93	98.32	92.62	98.31
FR_6	50.17	49.74	4.97	98.23	92.33	99.16
FS_2	50.17	49.71	4.97	98.14	92.03	99.08
FTR_7	50.17	49.69	4.97	98.18	92.14	99.05
FTS_3	50.17	49.44	4.94	98.25	92.53	98.54

CS1 (control susceptible), FS2 (*Fusarium* susceptible), FTS3 (*Fusarium Trichoderma* susceptible) and FBS4 (*Fusarium Bacillus* susceptible) for Mchare and CR5 (control resistant), FR6 (*Fusarium* resistant), FTR7 (*Fusarium Trichoderma* resistant) and FBR8 (*Fusarium Bacillus* resistant) for Grand Naine.

Supplementary Table 2. Summary of Genome Mapping

Sample	Total Clean Reads	Total Mapping Ratio (%)	Uniquely Mapping Ratio (%)
CR_5	49,669,582	27.27	19.92
CS_1	49,571,706	31.33	23.14
FBR_8	49,711,170	26.06	18.73
FBS_4	49,318,444	30.45	22.34
FR_6	49,744,462	29.92	21.71
FS_2	49,708,094	31.25	22.83
FTR_7	49,690,694	27.99	20.27
FTS_3	49,436,120	32.37	23.72

CS1 (control susceptible), FS2 (*Fusarium* susceptible), FTS3 (*Fusarium Trichoderma* susceptible) and FBS4 (*Fusarium Bacillus* susceptible) for Mchare and CR5 (control resistant), FR6 (*Fusarium* resistant), FTR7 (*Fusarium Trichoderma* resistant) and FBR8 (*Fusarium Bacillus* resistant) for Grand Naine.

Sample	SE	MXE	A5SS	A3SS	RI
CR_5	1,308	36	204	409	1,016
CS_1	1,305	39	186	384	964
FBR_8	1,424	53	208	389	973
FBS_4	1,399	45	217	427	992
FR_6	1,500	62	213	396	982
FS_2	1,379	50	211	407	993
FTR_7	1,446	49	206	399	991
FTS_3	1,297	43	206	409	984

Supplementary Table 3. SNP variant type summary (IR: intron retention, A3SS: alternative 3′ splice sites, A5SS: alternative 5′ splice sites, SE, skipping exon, AFE: alternative first exons, ALE: alternative last exons, CSE: coordinated skipping exons, MXE: mutually exclusive exons)

CS1 (control susceptible), FS2 (*Fusarium* susceptible), FTS3 (*Fusarium Trichoderma* susceptible) and FBS4 (*Fusarium Bacillus* susceptible) for Mchare and CR5 (control resistant), FR6 (*Fusarium* resistant), FTR7 (*Fusarium Trichoderma* resistant) and FBR8 (*Fusarium Bacillus* resistant) for Grand Naine.

Supplementary Table 4. SNP variant type summary

Sample	A-G	C-T	Transition	A-C	A-T	C-G	G-T	Transversion	Total
CR_5	146,763	146,414	293,177	49,251	62,089	40208	49,078	200,626	493,803
CS_1	103,564	103,936	207,500	34,834	42,155	30,483	34,663	142,135	349,635
FBR_8	116,301	116,714	233,015	38,730	47,659	32,917	38,674	157,980	390,995
FBS_4	120,238	120,035	240,273	40,208	49,354	34,312	40,224	164,098	404,371
FR_6	122,022	122,398	244,420	40,632	51,063	33,942	40,844	166,481	410,901
FS_2	114,896	114,882	229,778	38,148	47,056	32,696	38,259	156,159	385,937
FTR_7	124,904	124,773	249,677	41,021	50,117	35,016	40,999	167,153	416,830
FTS_3	105,723	106,507	212,230	35,246	42,648	30,717	35,101	143,712	355,942

CS1 (control susceptible), FS2 (*Fusarium* susceptible), FTS3 (*Fusarium Trichoderma* susceptible) and FBS4 (*Fusarium Bacillus* susceptible) for Mchare and CR5 (control resistant), FR6 (*Fusarium* resistant), FTR7 (*Fusarium Trichoderma* resistant) and FBR8 (*Fusarium Bacillus* resistant) for Grand Naine.

Supplementary Table 5. Summary of gene mapping ratio

Sample	Total Clean Reads	Total Mapping Ratio (%)	Uniquely Mapping Ratio (%)	Total Gene Number	Total Transcript Number
CR_5	49,669,582	31.67	21.03	37,710	37,710
CS_1	49,571,706	41.38	26.41	37,820	37,820
FBR_8	49,711,170	32.78	21.75	36,393	36,393
FBS_4	49,318,444	38.45	24.92	38,055	38,055
FR_6	49,744,462	37.47	24.99	35,966	35,966
FS_2	49,708,094	41.11	27.03	37,352	37,352
FTR_7	49,690,694	35.05	23.09	37,625	37,625
FTS_3	49,436,120	43.2	28.04	37,792	37,792

CS1 (control susceptible), FS2 (*Fusarium* susceptible), FTS3 (*Fusarium Trichoderma* susceptible) and FBS4 (*Fusarium Bacillus* susceptible) for Mchare and CR5 (control

resistant), FR6 (*Fusarium* resistant), FTR7 (*Fusarium Trichoderma* resistant) and FBR8 (*Fusarium Bacillus* resistant) for Grand Naine.

Supplementary Table 6. Genes and transcripts statistics

Sample	Total Gene Number	Total Transcript Number
CR_5	37,710	37,710
CS_1	37,820	37,820
FBR_8	36,393	36,393
FBS_4	38,055	38,055
FR_6	35,966	35,966
FS_2	37,352	37,352
FTR_7	37,625	37,625
FTS_3	37,792	37,792

CS1 (control susceptible), FS2 (*Fusarium* susceptible), FTS3 (*Fusarium Trichoderma* susceptible) and FBS4 (*Fusarium Bacillus* susceptible) for Mchare and CR5 (control resistant), FR6 (*Fusarium* resistant), FTR7 (*Fusarium Trichoderma* resistant) and FBR8 (*Fusarium Bacillus* resistant) for Grand Naine.
